# Controlled Manipulation and Active Sorting of Particles Inside Microfluidic Chips Using Bulk Acoustic Waves and Machine Learning

**DOI:** 10.1021/acs.langmuir.1c00063

**Published:** 2021-04-02

**Authors:** Kyriacos Yiannacou, Veikko Sariola

**Affiliations:** Faculty of Medicine and Health Technology, Tampere University, Korkeakoulunkatu 3, 33720 Tampere, Finland

## Abstract

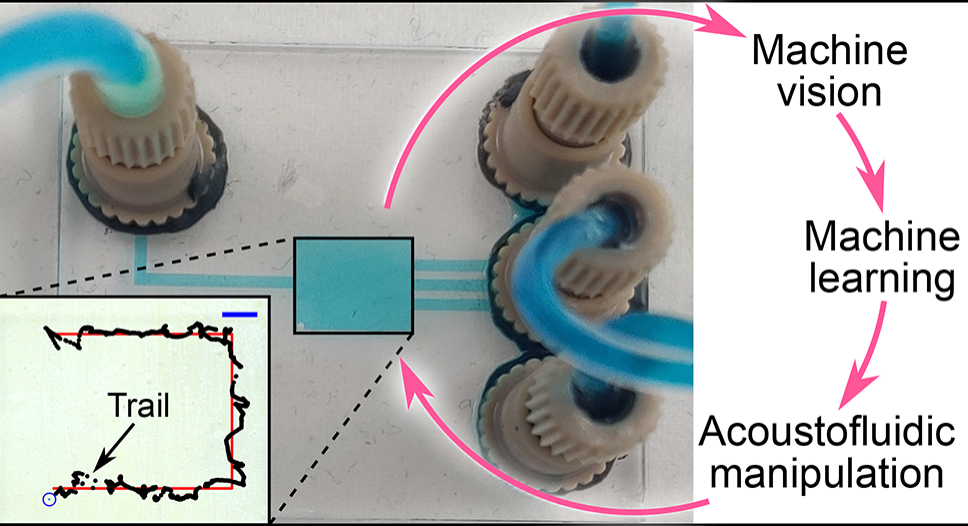

Manipulation of cells, droplets, and particles via ultrasound within microfluidic chips is a rapidly growing field, with applications in cell and particle sorting, blood fractionation, droplet transport, and enrichment of rare or cancerous cells, among others. However, current methods with a single ultrasonic transducer offer limited control of the position of single particles. In this paper, we demonstrate closed-loop two-dimensional manipulation of particles inside closed-channel microfluidic chips, by controlling the frequency of a single ultrasound transducer, based on machine-vision-measured positions of the particles. For the control task, we propose using algorithms derived from the family of multi-armed bandit algorithms. We show that these algorithms can achieve controlled manipulation with no prior information on the acoustic field shapes. The method learns as it goes: there is no need to restart the experiment at any point. Starting with no knowledge of the field shapes, the algorithms can (eventually) move a particle from one position inside the chamber to another. This makes the method very robust to changes in chip and particle properties. We demonstrate that the method can be used to manipulate a single particle, three particles simultaneously, and also a single particle in the presence of a bubble in the chip. Finally, we demonstrate the practical applications of this method in active sorting of particles, by guiding each particle to exit the chip through one of three different outlets at will. Because the method requires no model or calibration, the work paves the way toward the acoustic manipulation of microparticles inside unstructured environments.

## Introduction

Manipulation of cells, droplets, and particles via ultrasound within microfluidic chips, acoustofluidics, is a rapidly growing field, with applications in cell and particle sorting,^1^ cell patterning,^2^ blood fractionation,^3,4^ droplet transport,^5^ and enrichment of rare or cancerous cells.^6,7^ The nature of this manipulation is contactless, which is why it is suitable for so many biological applications.^8^ Furthermore, being contactless also simplifies the chip fabrication, because the transducers do not need to be in direct contact with the manipulated object. Integrating acoustic functionalities into microfluidic chips is a promising approach toward the realization of entire laboratories on chips.

Typically, such chips consist of structured chambers, ports, and closed channels, with dimensions ranging from a few micrometers to a few millimeters. The devices can be actuated via vibrations through the bulk (the whole chip or the chamber vibrated by a piezoelectric transducer) or by exciting surface acoustic waves along the surface of a piezoelectric substrate.^9^

Bulk acoustic wave devices usually use a single-frequency or a narrow-frequency band, and the channel widths are half of the acoustic wavelength. At half wavelength resonance, a standing wave develops between the walls of the channel, forcing the particles to migrate toward the walls (pressure antinode) or toward the middle (pressure node), depending upon their acoustic contrast factor relative to the medium.^10^

A potential application of such devices is in passive cell and particle separation, where continuous flow and an acoustic field transport particles or cells toward a particular outlet.^4,6,7^ The outlet through which a particle exits depends upon its acoustic contrast factor and its size/shape. For example, red blood cells can be separated from a contaminated solution,^6^ or mononuclear cells can be separated from whole blood.^11^ Particle manipulation,^12,13^ pattern formation,^14^ microassembly,^15^ and particle trapping^16^ based on acoustophoresis have also been reported.

As an example of a more controlled acoustofluidic manipulation, alternating between two frequencies, which correspond to two different resonances of a channel, has been used to deterministically drive a single droplet to either of two different outlets.^1^ Thus far, similar methods have been mostly limited to binary control actions (field on/off), frequency modulations,^17^ multi-frequency switching to create unique cell aggregation patterns, or at most a few frequencies.^18−21^ A higher degree of control is usually achieved using multiple transducers to carefully construct the desired field shape: holographic traps^22^ or perpendicular surface acoustic wave (SAW) actuators.^20,23^ Deep learning has been used to relate the geometry of the manipulation area to the observed acoustic fields.^24^ This method was able to predict and produce accurate acoustic field models for a variety of channel shapes.

Recently, our co-authors demonstrated a method for the controlled manipulation of particles on a flexurally vibrating thin plate (so-called Chladni plate).^13^ The method is based on the extensive modeling of the particle motion in response to different frequencies. The manipulation is performed by choosing which frequency to play next based on the current position of the particles and where one wants them to go in a closed-loop manner. The method has since then been extended to Chladni plates submerged in a liquid medium^25^ and different control algorithms.^26,27^ Because the plates were 50 × 50 mm and the waves were flexural, the frequencies used were well in the audible range.

**Figure 1:**
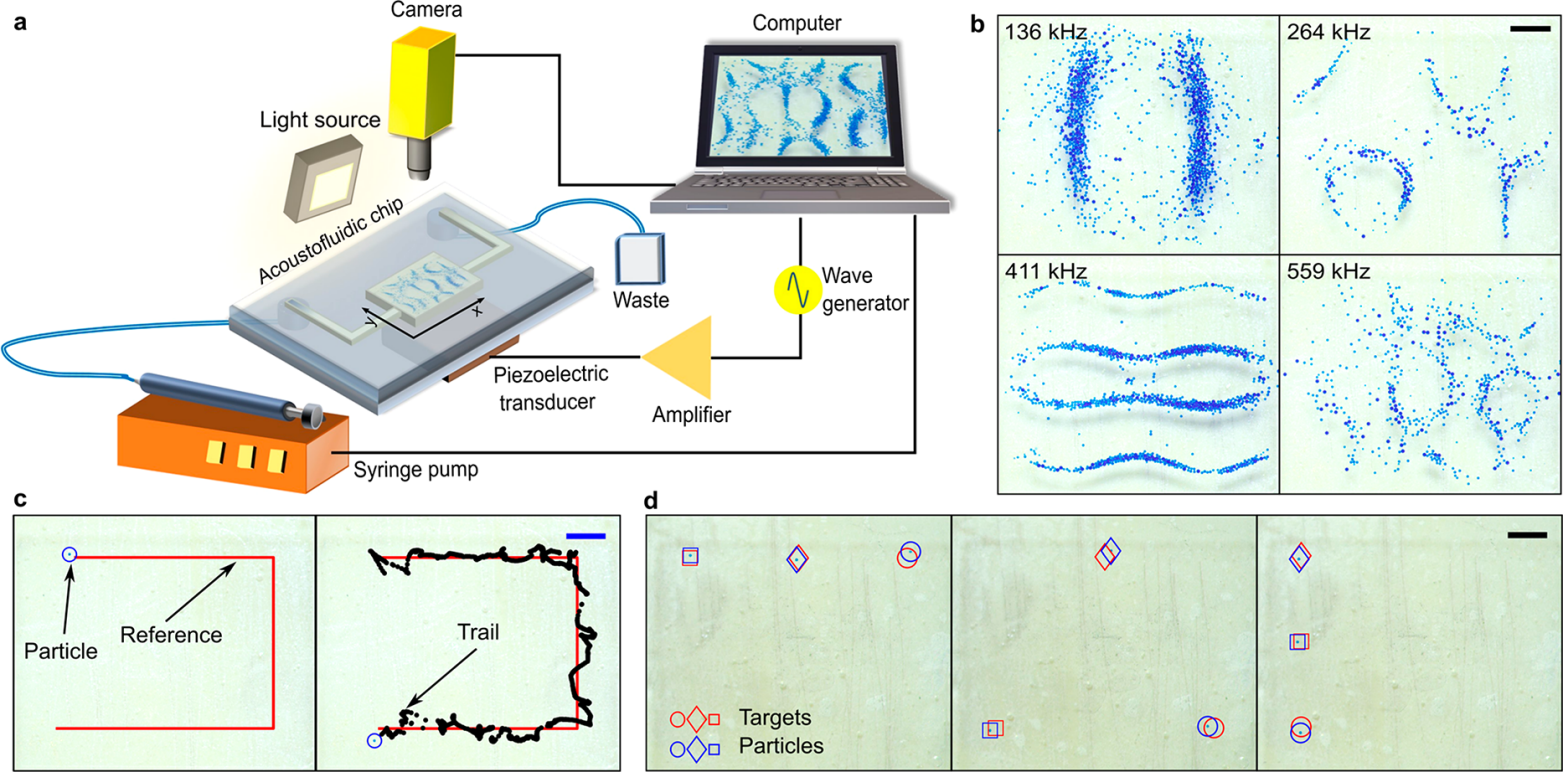
Controlled acoustofluidic manipulation using bulk acoustic waves inside a microfluidic chip. (a) Schematic of the experimental setup. (b) Acoustic patterns for various frequencies. (c) Single-particle manipulation, with the line following a single particle manipulated using bulk acoustic waves. (d) Multiparticle manipulation. All scale bars = 1 mm.

In this paper, we demonstrate the closed-loop two-dimensional (2D) manipulation of particles (Figure 1) inside closed-channel microfluidic chips, by controlling the frequency of a single ultrasound transducer, based on machine-vision-measured positions of the particles. Closed-channel microfluidics poses a new problem for such an acoustic manipulation: it is difficult to perform controlled calibration or learning experiments because one cannot manually place a particle at any given location inside the chamber. To overcome this problem, in this paper, we show that algorithms derived from the well-known family of multi-armed bandit algorithms can achieve controlled manipulation with minimal learning, voltage adjustments, or reinforcement learning. The method “learns as it goes”: there is no need to restart the experiment at any point. Starting with no knowledge of the acoustic field shapes, the algorithm can (eventually) move a particle from one position inside the chamber to another. The multi-armed bandit algorithms determine how to move the particles solely based on the information that they have accumulated about the device starting from the beginning of each particular experiment. This method is highly agnostic to the actual acoustic manipulation system: it knows nothing about the acoustic field shapes or actual frequencies, just that there are *N* frequencies that could be used. Thus, it should be relatively straightforward to adapt it to other acoustic manipulation systems, including, e.g., the Chladni plate setup from the previous work of our co-authors. We also demonstrate that the method can be adapted to the manipulation of multiple particles, and we demonstrate the practical applications of this method in the active sorting of particles into one of the three outlets of the chip.

In short, we present a method for manipulating microparticles inside microfluidic chips by controlling the frequency of a single ultrasound transducer based on machine-vision-measured positions of the particles and machine learning. This method is robust to changes in chip/sample and object properties, such as changes in the particle size, with the potential trade-off of longer manipulation times. Because the method requires no model or calibration, the work paves the way toward the acoustic manipulation of microparticles inside unstructured environments.

## Experimental Section

Two different glass chips were designed, one with a single outlet and another one with three outlets (panels a and b of Figure S1 of the Supporting Information). The single-outlet chip was designed for simple manipulation tests, while the multi-outlet chip was designed for demonstrating sorting of particles into one of the outlets. Both chips have a single inlet. The chips include a rectangular manipulation chamber (length, 7 mm; width, 6 mm; and height, 0.15 mm). The dimensions of the rectangular chambers were kept the same in both chips, so that the manipulation is performed roughly in the same frequency range. The dimensions of the chamber were chosen to be asymmetric on purpose, to separate width and length modes. After deciding the dimensions of the chamber, we calculate the overall lateral dimensions of the chip to facilitate a quarter wavelength acoustic wave in the glass edges and a half wavelength wave in the chamber.^28^ The chip dimensions were calculated using the values in Table 1.

**Table 1:** Material Parameters

material	speed of sound (m s^–1^)	density (g cm^–3^)
fused silica glass	5968	2.2
water	1487	1

This method has been reported to enhance the strength of acoustic resonances inside the chamber.^28^ Note that this chamber size not only supports half-wavelength resonances but also higher harmonics.

The chips were fabricated by wet etching of fused silica glass. All inlet and outlet channels were 1.1 mm wide and 0.15 mm deep. Nanoport (IDEX Health & Science, LLC) fluidic connectors were glued to drilled inlets and outlets. The microfluidic chips were fabricated by Klearia, France. A 15 × 15 × 2 mm (NCE45, Noliac, Denmark) piezoelectric transducer was glued (Figure 1a) on the bottom of the chip, approximately in the center, using epoxy glue (Loctite Power Epoxy). Panels c and d of Figure S1 of the Supporting Information show the fabricated chips.

As a starting point for selecting the frequency range for manipulation, we calculated the expected resonance frequencies by assuming infinite hard walls on the water–glass interface, given the following equation:^28^
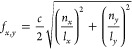
1where *c* is the speed of sound in water, *l*_*x*,*y*_ are the length and width of the chamber, respectively, and *n*_*x*,*y*_ are the mode numbers.^28^ This gives *f*_1,0_ = 106.2 kHz as the first resonance in the length direction and *f*_0,1_ = 123.9 kHz as the first resonance in the width direction.

### Experimental Setup

The experimental setup includes a radio frequency (RF) amplifier to drive the piezoelectric transducer, a camera to image the manipulation chamber, a light source, a computer to implement the closed-loop control, and a syringe pump to deliver the particle solution into and out of the chamber. A schematic of the experimental setup is shown in Figure 1a.

In the particle manipulation experiments, ≈70 μm polystyrene particles (Lab261, Palo Alto, CA, U.S.A.; density, 1.05 g/cm^3^; and color, blue) were used. To prevent the particles from agglomerating as a result of their hydrophobic behavior, we used 1 vol % Triton X-100 in deionized (DI) water as the medium.

The fluid–particle suspension was delivered to the chip by a syringe pump (Aladdin, World Precision Instruments) at a rate of 0.01 mL/min. The particles exiting from the outlet of the chamber were captured into an output reservoir. Silicone tubing was used for all fluidic connections.

The piezoelectric transducer was excited by an electrical signal from a computer with an embedded waveform generator (PCI-5412, National Instruments). The signal was amplified by a 400 W class AB RF amplifier (1400L, Electronics & Innovation).

The particles were imaged with a camera (Basler acA2040-120uc, Germany), and their *x*/*y* position was tracked by a custom machine vision algorithm, written in MATLAB. To enhance the visibility of the particles within the chamber, a 100 W rectangular light-emitting diode (LED) was used to illuminate the chamber.

### Closed-Loop Control Algorithms

The control algorithms tested in this work are UCB1^29^ and ε-greedy^30^ from the multi-armed bandit family of algorithms. As a control experiment, the linear programming control algorithm from the previous work of our co-authors was also used.^13^ The pseudocode for the control algorithms is given in Supplementary Note 1 of the Supporting Information, and our implementation can be downloaded from Zenodo.^31^

Briefly, the task of the control algorithm is to choose which frequency to play next. In both control algorithms, the frequencies are discrete: the algorithms choose one of the *N* = 100 frequencies, linearly spaced in the frequency range from 65 to 700 kHz. The chosen frequency is played for half a second, and the algorithms then assign a reward for the action, with the reward being simply how many pixels or micrometers the particle moved toward its current target point. In subsequent rounds, the multi-armed bandit algorithms balance between exploration and exploitation: playing the frequencies that gave the largest (average) rewards in the past versus trying out new frequencies that could be even better.

Exactly how exploration and exploitation is balanced differs in the UCB1 and ε-greedy algorithms. In ε-greedy, at each step, any random frequency is chosen with a probability of ε (exploration). Otherwise (with a probability of 1 – ε), the frequency with the highest average past reward is chosen (exploitation). In UCB1, a confidence interval around the mean is computed, where the magnitude of the confidence interval is ∼, where *n* is the total number of actions taken and *n*_*j*_ is the number of times action (frequency) *j* has been chosen. The frequency with the highest upper confidence bound (mean + confidence) is chosen. The effect of this confidence bound is that the algorithm is “optimistic”: a poor performance of a frequency with only a few trials might be due to bad luck (exploration). As evidence accumulates (a frequency is played many times), the confidence interval decreases and the algorithm will start to choose solely based on average past rewards (exploitation).

The rewards vary with the current position and the target position of a particle: a frequency that is good for moving toward a node is not good for moving out of that node. For this reason, the average rewards were computed using exponentially decaying weights; i.e., the weights were proportional to γ^–*t*^, where γ is the weight factor and *t* is the number of control steps since that reward. This exponentially decaying weight is also taken into account when calculating the confidence bound for the UCB1 algorithm: the algorithm slowly forgets having chosen a particular frequency, and the confidence bound slowly increases again. This exponentially decaying memory ensures that old rewards, not anymore relevant to the current position and target of the particle, are forgotten.

### Adjusting the Excitation Amplitude for Different Frequencies

In the previous work on Chladni plates, the driving amplitude of the transducer was adjusted for each frequency, so that the median motion of particles was approximately the same for all frequencies.^13^ The purpose of this process was to avoid excessive motion of the particles when driving the system near resonances and also to detect the locations of the resonances of the system.^13^ We conducted similar experiments with our system. For these experiments, approximately 600–1000 particles were pumped into the chamber. Starting with a voltage of 30 V for each frequency, several rounds of experiments were conducted, and in each round, every frequency was excited once. After each experiment, the displacement of all particles was recorded by the camera and analyzed using MATLAB and the median displacement was computed. Dependent upon the median displacement, the voltage for that frequency was increased or decreased, depending upon whether the displacement was larger or smaller than a defined threshold.

**Figure 2:**
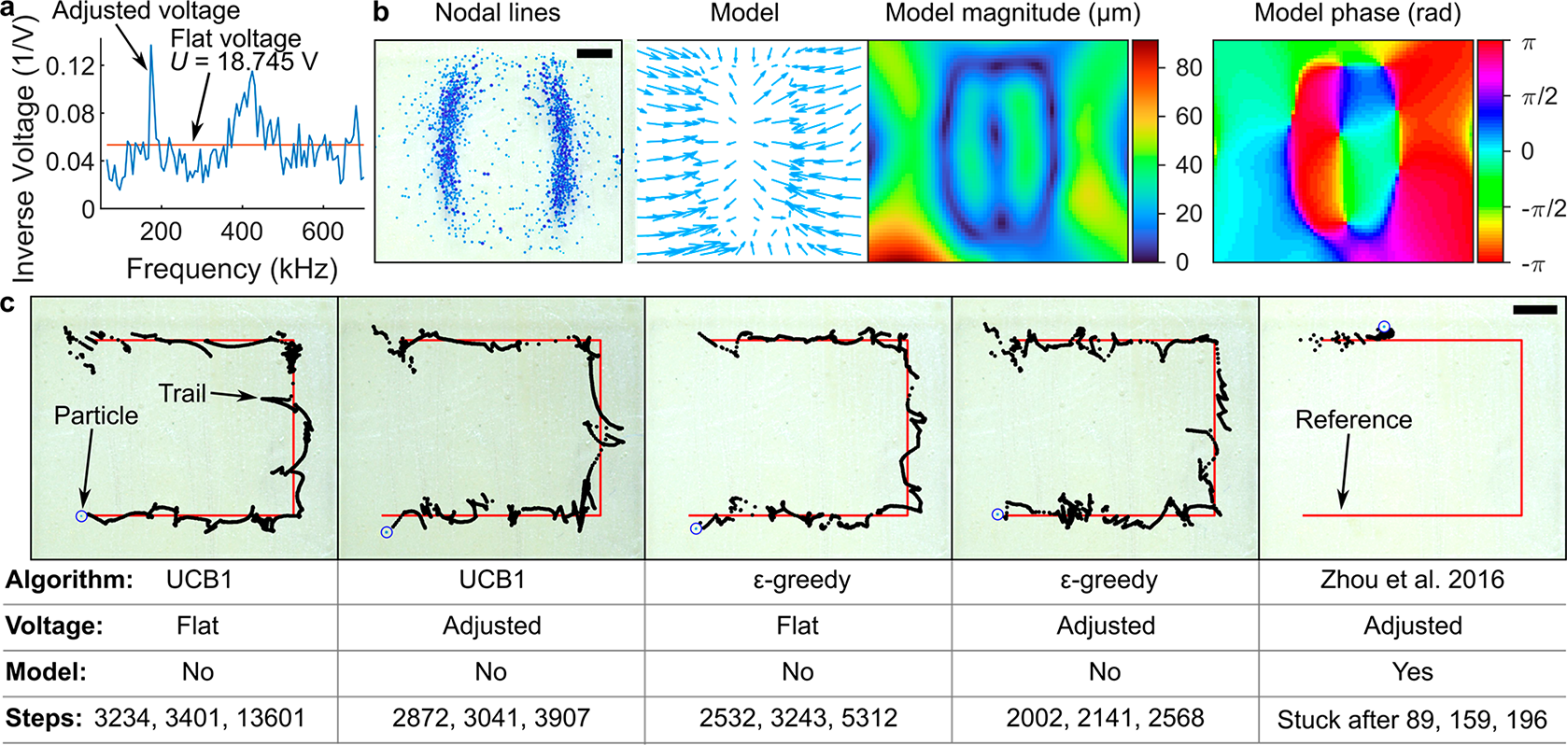
Comparison of different controllers for acoustofluidic manipulation. (a) Driving voltages for different frequencies, after adjusting them, so that they result in a motion of 25 pixels for a 0.5 s signal. (b) Comparison between Chladni figures and the data-driven models that predict how the particles move at 136 kHz. (c) Comparison of different controllers for the manipulation. Flat voltage means that a voltage of 18.745 V was used for all frequencies, and adjusted voltage means that the voltage was chosen according to panel a. Model means that the algorithm needs the calibration models from the experiments, exemplified in panel b (model “no” means that no data-driven models of the acoustic field shapes were used). Steps is the number of control steps taken in three different experiments; each step ≈ 1 s (the frequency is played for 0.5 s; machine vision and control computations take another ≈0.5 s). All scale bars = 1 mm.

In our case, the threshold was set to 25 pixels ≈ 173 μm. This process generated data that relate particle motion with the resonances of the acoustic device. When the acoustofluidic chip is driven at or near resonances, lower voltages were needed to achieve the desired median displacement. We study the resonances by plotting the inverse of the voltage against the applied frequency (Figure 2a).

### Modeling of the Displacement Fields

To compare our work to the earlier work from the literature, we also tested using the linear programming controller from the previous work of our co-authors. This linear programming controller required the knowledge of the field shapes for each frequency. To record these field shapes, we followed their approach.^13^ Briefly, >500 particles were pumped to the chamber, and then all of the discrete frequencies were excited 5 times, in random order. Between every frequency played, the particles were withdrawn from and pumped back to the chamber to distribute the particles evenly inside the chamber and to break particle clusters.

The particle positions **p** and particle displacements Δ**p** were captured by machine vision after exciting one frequency. This resulted in a data set of (**p**, Δ**p**) for each frequency. From the captured data points, the displacement values |Δ**p**| > 350 μm were discarded, because they represent false detections or wrong matching of particles by the machine vision. For each frequency, we estimate the two-dimensional displacement field by performing robust LOESS regression.^13,32^ An example of the resulting model for a frequency of 136 kHz is shown in Figure 2b.

## Results and Discussion

### Single-Particle Manipulation

To show that we can actively guide a particle inside the single-outlet chip using the multi-armed bandit-based controllers (ε-greedy and UCB-1), we defined a U-shaped reference path for a particle to follow. During the manipulation, a point along the reference path (waypoint) serves as a temporary target point. As the particle approaches the waypoint within 300 μm, a new waypoint along the path is chosen as the target point. The results for such manipulation experiments are shown in Figures 1c and 2c. Both multi-armed bandit controllers were able to successfully guide the particle to follow the given reference trajectory, showing that a controlled acoustofluidic manipulation is possible without any prior learning events or acoustic field models.

The accuracy of the multi-armed bandit controllers was limited by the chosen waypoint tolerance (300 μm in our experiments). This is a trade-off with the manipulation time: a smaller tolerance value increases the accuracy but also increases the manipulation time. Besides tuning the tolerance value, one could also reduce the voltage, so that the particles move less and avoid large jumps around the reference trajectory. Furthermore, the manipulation time varies randomly between each run (Figure 2c). This is not surprising given the chaotic nature of the manipulation: small differences in the initial position of a particle and the experimental conditions can lead to a very different path taken. Also, in the case of the ε-greedy algorithm, the algorithm itself is stochastic.

We did not observe any obvious heat-induced effects in any of the experiments. Even the longest experiment, which lasted approximately 3 h (∼14 000 steps), we did not see any evidence of the chamber heating (boiling and particle melting). We conclude that our method can be used to perform long-term manipulations without being limited by thermal effects.

As a result of the long duration of the experiments, the polystyrene particles are expected to sediment to the bottom of the chamber. The terminal velocity of our particles, calculated by balancing Stokes’ drag to gravitational effects (taking buoyancy into account), is ∼0.1 mm/s. Our experiments last up to tens of minutes; therefore, within the time frame of a single experiment, the particles are expected to sediment to the bottom. Because the frequency range in this work was kept well below the resonance frequencies of the vertical modes, the vertical modes are unlikely to create enough force to counter the sedimentation.

To show that our method is not limited to manipulating particles of a certain size, we repeated the same single-particle manipulation experiments as in Figure 2c with a 100 μm diameter particle. The results are shown in Figure S4 of the Supporting Information. The controller was successful in guiding the particle along the refence path. The experiment lasted ∼40 min with a total of 2593 control steps. These results show that our manipulation method is not limited to particles of a specific size. In our setup, a hard upper limit for the particle size comes from the dimensions of the chip: the height of the chamber is 150 μm. A hard lower limit comes from the resolution of our imaging system: each pixel is 6.9 μm.

To show that our method is robust to the variations between one chip and another, we redid the single-particle manipulation experiment with our multi-outlet chip, using the same frequency values and a constant voltage of 20.64 V. The particle was successfully guided within the chip (Movie S1 of the Supporting Information), despite the exact shape of the chamber being different from our single-outlet chip. This further confirms that our method is not limited to the specific chip design and is robust to changes in chip geometry.

Bubbles are a common issue in microfluidic systems, and their presence could significantly distort the acoustic field shapes, potentially impairing acoustofluidic manipulation systems. To show that our method is robust to the presence of air bubbles, we performed a simple path following the experiment with a large bubble in the chamber. Despite the bubble, the controller was able to successfully guide the particle through the path (Movie S4 of the Supporting Information). We attribute this to the short memory of the algorithm: it can quickly adapt to the disturbances from the bubbles, whereas in future steps, the algorithm equally quickly forgets the existence of the bubbles.

Whereas the multi-armed bandit controllers were able to complete the manipulation tasks, the previously reported linear programming controller^13^ failed to complete the manipulation tasks. We attribute these failures to errors in the displacement field models used by the linear programming controller. Because the controller is non-stochastic and non-adaptive, the controller got stuck in an infinite loop when it thought that the particle should move in response to a frequency, but it did not. We suggest that these errors are from the difficulty of distributing the particles evenly inside the chamber: if very few or no particles were near position **p** during calibration experiments, the model cannot be used to predict Δ**p**. Figure S2 of the Supporting Information shows an example of how the particles were distributed during the calibration experiments.

As a concrete example of the resulting modeling errors, see Figure 2b. In the bottom left corner, there is a bright red spot in the magnitude plot, indicating displacements of over 80 μm. This is most certainly an error, as we observed almost no particles near the corners during modeling experiments, because there are no nodes in the corner of the chip.

More displacement field models can be found in Figure S3 of the Supporting Information. Some of the patterns are symmetric, but most are not, even though a simplistic model of a symmetric chamber with a piezo mounted in the center says that they should be. This is a well-known problem in acoustic manipulation: even slight misalignments could cause the antisymmetric modes to be excited. We take this as an evidence that it would also be difficult to derive these displacement fields from first principles. In summary, the major problem in the linear programming controller is that it needs accurate models of the displacement fields, and in closed-channel microfluidics, it is difficult to obtain these models either using data-driven techniques or from first principles.

To further compare the discrepancies between theoretical and experimentally obtained mode shapes, one can try to correlate the resonance frequencies predicted by eq 1 to the practically observed field shapes in Figure S3 of the Supporting Information. For example, in Figure S3 of the Supporting Information, we find a shape resembling a half wavelength mode *f*_1,0_ at 129 kHz, while the simplistic model of eq 1 predicts *f*_1,0_ = 106.2 kHz, ∼23 kHz lower than the experimentally observed value. Plausible reasons for the discrepancy include inlets, uncertainties in the physical parameters (for example, the effect of Triton X-100 on the speed of sound or the actual speed of sound in the glass or water), and manufacturing tolerances in the chip. To conclude, the unpredictability of the experimentally obtained acoustic field shapes highlights why our proposed adaptive, machine-learning-based control techniques are useful for acoustofluidic manipulation.

### Multiparticle Manipulation

To show that our method can be extended to multiparticle manipulation, we tested manipulating three particles simultaneously in the single-outlet chip using the ε-greedy control algorithm. This manipulation task is complicated and challenging because the particle motion in the manipulation space is coupled.^22^ However, our method is trivial to extend to multiparticle manipulation: when calculating the rewards, we simply sum the rewards from each of the three particles. The controller manages to uncouple the particle motion and individually move the particles to their target points. Figure 1d presents the multiparticle manipulation steps, where initially the particles from different parts of the chamber were driven to the targeted locations. Once the particles reached the initially assigned targets, a new set of targets was then assigned. This process was continued until the particles reached the final target locations. The algorithm managed to uncouple the motion of the three particles within the chamber and individually guide them toward the selected target points (Movie S2 of the Supporting Information).

In multiparticle manipulation, the control of individual particles may become difficult if they come too close to each other. There are three reasons for these difficulties: (1) The primary acoustic forces of the two particles become highly correlated: the wavelength of the highest frequencies is longer than the distance between the particles; therefore, the motions of the particles are highly correlated for all manipulation frequencies. This has been observed for other acoustic manipulation systems also.^13^ (2) The secondary acoustic force tends to drive two particles toward each other: in acoustofluidic manipulation systems, the particles themselves affect the acoustic field surrounding them, and the interaction between multiple particles in the vicinity of each other tends to agglomerate the particles.^33^ (3) Finally, our machine vision system can lose the tracking of individual particles, if they come too close to each other, especially if they touch each other.

In our multiparticle manipulation experiments, we avoided all of the aforementioned difficulties by keeping all particles far away from each other, by keeping the target points of particles far enough from each other. Typically, the distance between two target points was at least 2.5 mm.

### Active Sorting of Particles

**Figure 3:**
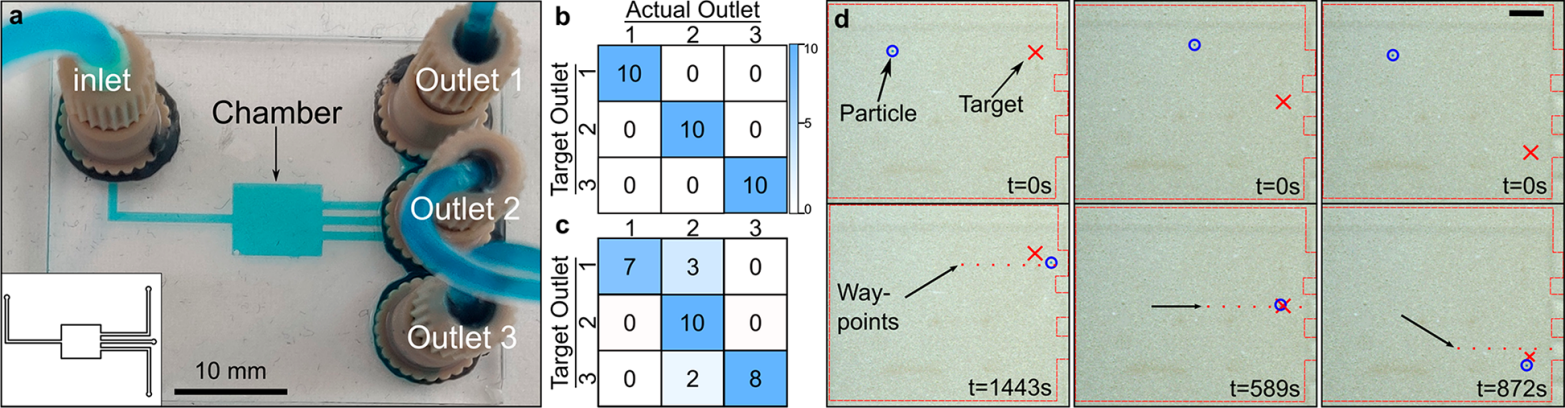
Particle-sorting experiments. (a) Photograph of the particle-sorting chip. The chip consists of a single inlet and three outlets. Inset shows the schematic of the chip. (b) Confusion matrix of particle-sorting experiments, with multiple waypoints leading to each outlet. (c) Confusion matrix of particle-sorting experiments, with a single way point, placed right at each outlet. (d) Snapshots of actual particle-sorting experiments. Scale bar = 1 mm.

To demonstrate a practical application of our manipulation method, we performed active particle sorting using the multi-outlet chip (Figure 3a). For each incoming particle, we assigned it to one of the three outlets, performed the manipulation experiment, and finally calculated the confusion matrix (assigned versus actual outlet) to characterize the reliability of the method. In the first sorting experiment, we sorted 30 particles, and the results are presented in Figure 3b. The controller was set to guide incoming particles through a conservative route. The conservative route was composed by several “guiding” waypoints, starting from a point close to the center of the chamber and ending at the selected outlet. The guiding waypoints used in these experiments are presented in Figure 3d. As seen from Figure 3b, no particle was missorted. In these experiments, the average sorting time per particle was 20 min.

We also tested faster particle sorting by not having any guiding waypoints but only having a single target point at the outlet of choice. The results are summarized in Figure 3c (see also Movie S3 of the Supporting Information). The average sorting time per particle decreased to 13 min, which is on average 7 min faster than the conservative sorting approach. However, this comes with a trade-off in sorting accuracy (Figure 3c): some of the particles are now missorted. These missortings are due to the controller: without guiding waypoints, the controller might accidentally drive the particle close to a wrong outlet, and as a result of the presence of a slight negative pressure at a wrong outlet, the particles accidentally exited through the wrong outlet. We conclude that the guiding waypoints maximize the sorting accuracy while only slightly increasing the sorting time per particle, whereas the single target approach suffers from particle missortings but is slightly faster.

## Conclusion

In this study, we showed that particles can be controllably manipulated and sorted within closed microfluidic chambers using bulk acoustic waves. The control algorithms were derived from the family of multi-armed bandit algorithms, a class of well-known machine learning algorithms. The manipulation was achieved by controlling the frequency of a single piezoelectric transducer. Even using a single transducer, the method could be used to manipulate three individual particles simultaneously.

While, in this study, the acoustic manipulation was demonstrated in 2D, adapting the algorithm itself to perform a three-dimensional (3D) manipulation would be very easy: when calculating the rewards (how many micrometers a particle moved toward its target), one would just calculate the distance of a particle to its target in three dimensions instead of two. The multi-armed bandit algorithm itself knows nothing of coordinates nor dimensions. 3D manipulation would necessitate tracking the particles in 3D; optical systems for tracking particles in *Z* direction in acoustofluidic context have been reported by Barnkob et al.^34,35^

One obvious potential future application of our method is in the manipulation of biological cells. Our particle sizes (70–100 μm) start to be in the upper range of mammalian cells: for example, adipocytes (fat cells) are approximately 100 μm. However, typical mammalian cells are in the range of 10–100 μm; thus, there is still ∼1 order of magnitude of scaling ahead of us. Cells are well-known to possess sufficient acoustic contrast to respond to bulk acoustic fields; thus, there are no fundamental obstacles why our method could not be used with biological cells. However, practical challenges include improving the imaging and particle tracking system and scaling down the chamber dimensions while increasing the manipulation frequencies.

There are a few hyperparameters for the algorithm, but these are limited in number: *N* (the number of frequencies), γ (the forgetting factor), and ε/*c* (the balance between exploration and exploitation, for ε-greedy or UCB1 algorithms, respectively). For the practical manipulation system, the minimum frequency, maximum frequency, and voltage are also parameters that can be adjusted. We have merely reported a combination of parameters that work; however, in the future, a more exhaustive mapping of the effects of various parameters on the manipulation speed and accuracy should be performed. For specific applications, the number of parameters in our control method is so low that optimizing them for a particular application should be feasible.

The advantages of our approach are as follows: (a) Model-free nature of our controllers: Our method requires absolutely no prior knowledge of the system acoustic fields, which can be expensive/tedious to measure accurately. (b) Easy to implement with a very few parameters: one problem that plagues machine learning is that the algorithms can be so complex and opaque that analyzing what they are actually doing is very difficult. For example, it is often difficult to know exactly what each layer of a deep-learning neural network is actually doing, whereas simple algorithms, like the multi-armed bandit algorithms here, can be written in 10 lines of code and are easy to monitor. (c) It is highly adaptable to variations in chips, fluid properties, and particle sizes: The amnesiac nature of our algorithms helps that it can quickly adapt to new conditions. We have demonstrated that they work in different chip designs (single-outlet versus multi-outlet chip) and can work in the presence of disturbances to the system (bubbles). This paves the way toward acoustofluidic microrobots, navigating particles through complex, unstructured environments.

The disadvantages of our method are the long manipulation times, which are a consequence of the memory of the algorithm being very short: it gains no speed from prior experiments. In its current incarnation, the long sorting times would limit the practical applications of our method to methods where only a few particles/cells are being manipulated, e.g., manipulation of oocytes, where one could expect to manipulate even single cells. In the future, we aim to develop the algorithms to balance the long and short memory: the algorithm could use prior information (long memory, e.g., using acoustic field shape models to predict how the particles move in response to a particular frequency) when it seems to give accurate results but can also switch to a more adaptive strategy (short memory) if the prior information proves misleading. Even with the current algorithms, the long sorting times could be improved in the future by switching between frequencies more rapidly and running the control/machine vision loop at a higher frequency.^36^

## Supporting Information Available

Movie showing single-particle manipulation (Movie S1) (MP4)
Movie showing multiparticle manipulation (Movie S2) (MP4)
Movie showing sorting of the particle (Movie S3) (MP4)
CAD designs and images of single-outlet and multi-outlet chips (Figure S1), distribution of particles resulting from pumping actions (infuse–withdraw) (Figure S2), modeled acoustic fields of the 100 frequencies use in the experiments (Figure S3), single-particle manipulation experiment with a 100 μm particle (Figure S4), and ε-greedy and UCB1 algorithm formulations (Supplementary Note 1) (PDF)
Movie showing single-particle manipulation in a chamber with air bubbles (Movie S4) (MP4)
