# Supplementary material for: Controlled Manipulation and Active Sorting of Particles Inside Microfluidic Chips Using Bulk Acoustic Waves and Machine Learning
Source: Langmuir. 2021 Apr 2;37(14):4192–9. doi: 10.1021/acs.langmuir.1c00063 (PMC8154862; doi:10.1021/acs.langmuir.1c00063)
Supplement: Supplementary file 4 — la1c00063_si_004.pdf [file la1c00063_si_004.pdf]

# Controlled Manipulation and Active Sorting of Particles Inside Microfluidic Chips Using Bulk Acoustic Waves and Machine Learning

*Kyriacos Yiannacou*[*a] and Veikko Sariola*[*a]*

Faculty of Medicine and Health Technology, Tampere University, Korkeakoulunkatu 3, 33720 Tampere, Finland

**Supporting Information**

**Number of Pages: 8**
**Number of Figures: 4**
**Number of Tables: 0**

**Supplementary Figures**

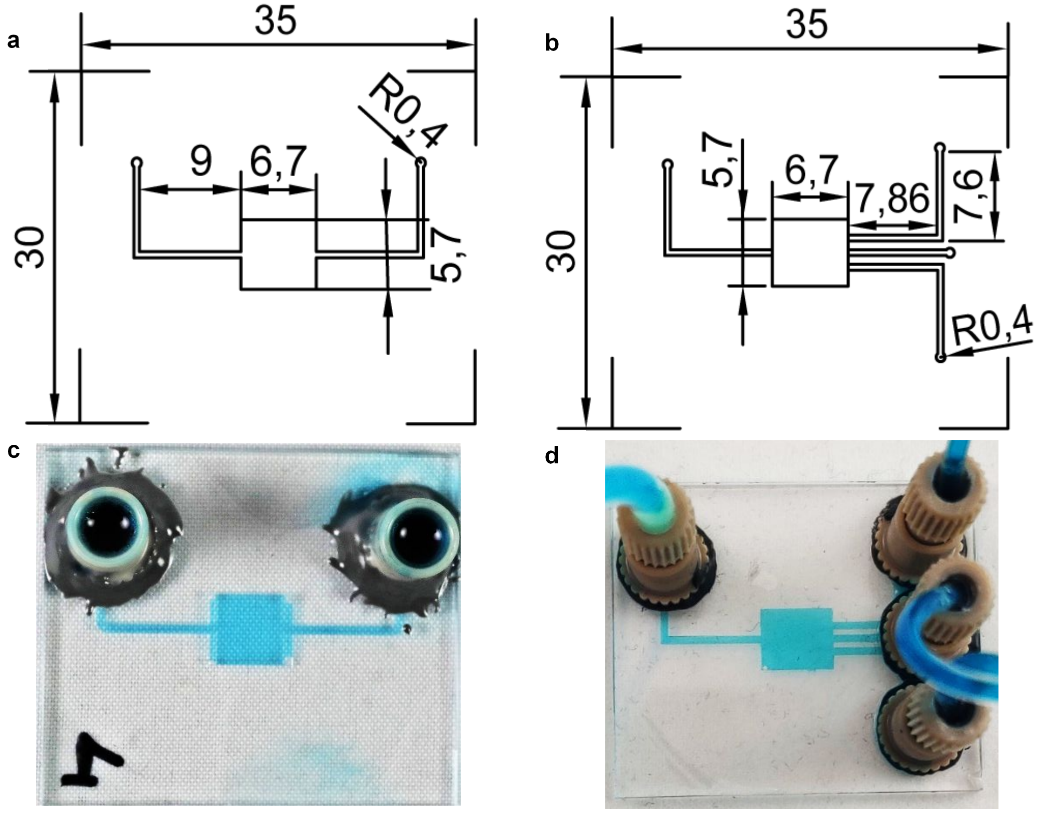


**Figure S1.** Single-outlet and multi-outlet chips. The CAD designs of the a) single-outlet and b) multi-outlet acoustofluidic chips. Fabricated c) single inlet-outlet and d) multi-outlet chips, prior to mounting the piezoelectric transducer.

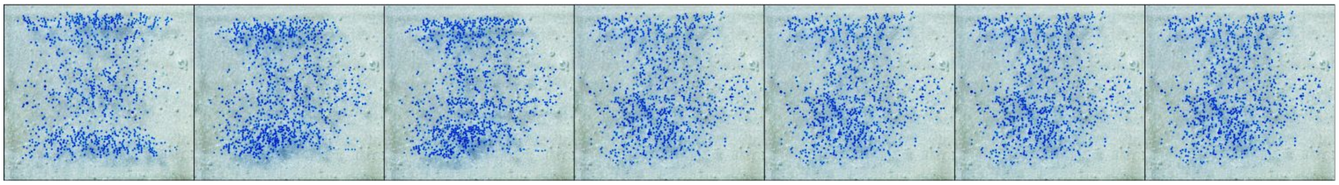

**Figure S2.** Distribution of particles, occurred from the infusing and withdrawing action of a syringe pump, prior actuation of each frequency during modelling experiments. Note that the particles are not evenly distributed throughout the chamber.

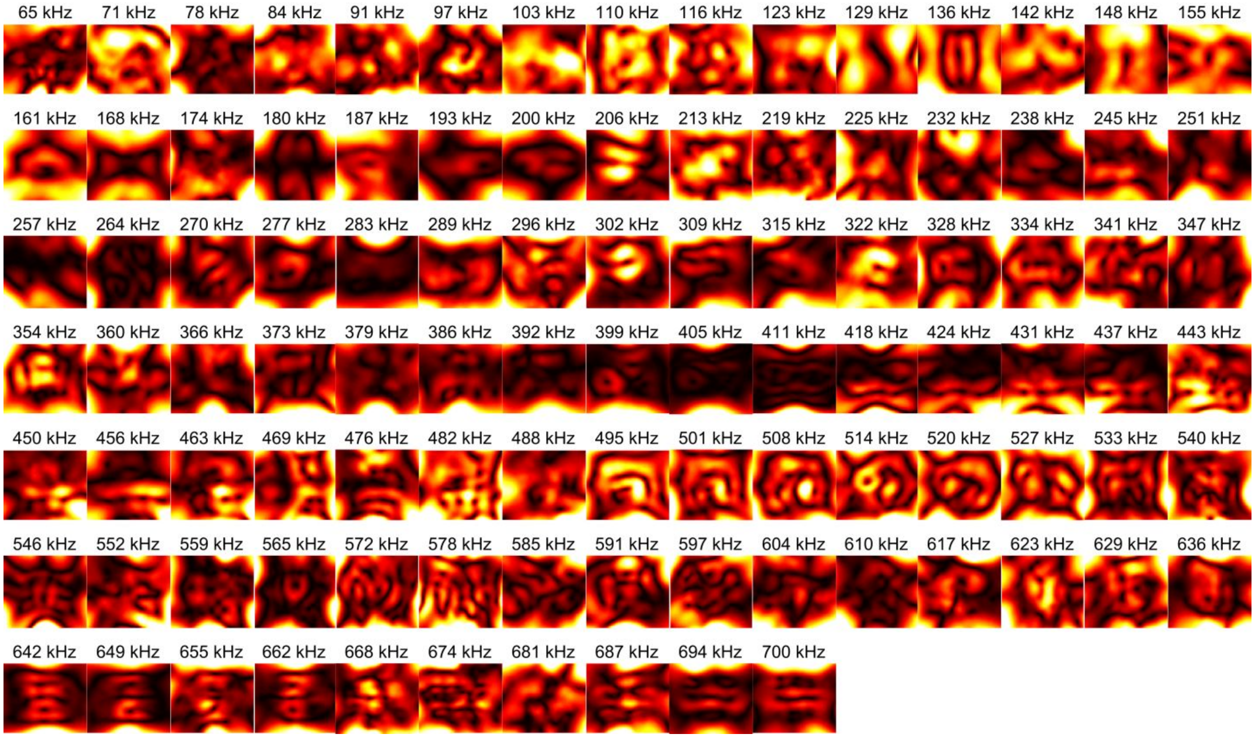


**Figure S3.** Acoustic field models, representing the particle displacement for every actuated frequency. The number of actuated is 100 frequencies, starting from 65 kHz and ending at 700kHz. The units of the colour maps are arbitrary, as the displacements are normalized for each frequency. Brighter colours correspond to larger motion.

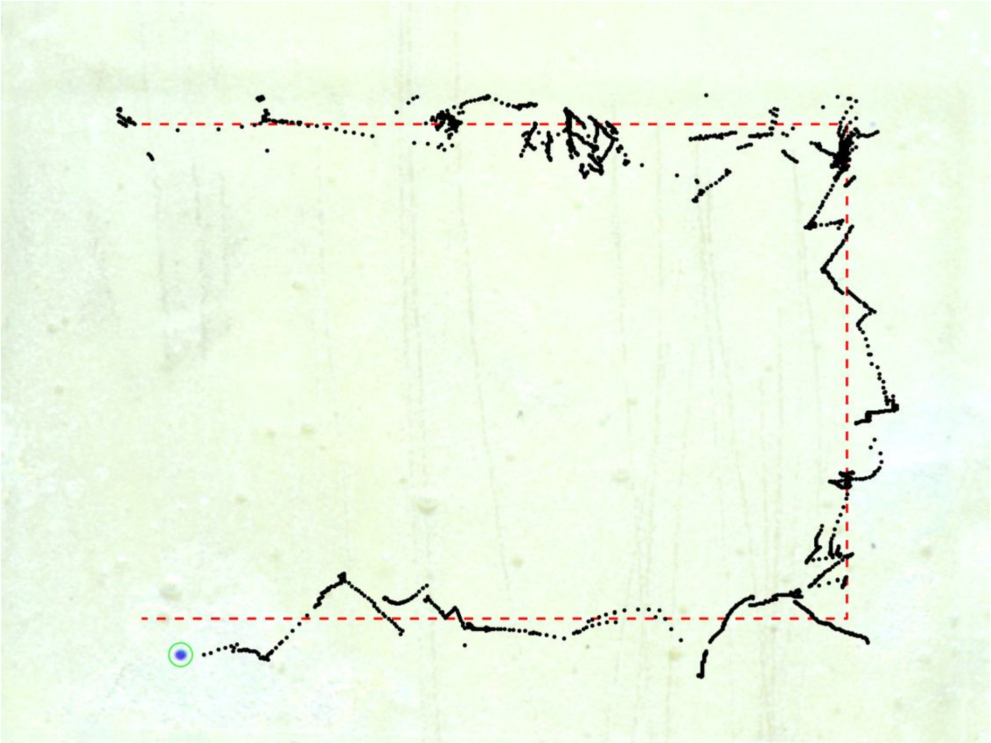

**Figure S4.** Single particle manipulation. Manipulation of 100 μm polystyrene particle, using ε-greedy controller.

**Supplementary Movies**

**Movie S1** Single-particle manipulation video. The video demonstrates single particle manipulation, performed in both the single-outlet and the multi-outlet chip. In both cases the ε-greedy control algorithm was used to manipulate the particle. Similar manipulation experiments are shown in Figs. 1c&2c.

**Movie S2** Multi-particle manipulation video. This video demonstrates the ability of our control method to manipulate three polystyrene particles simultaneously and guide them towards their target locations. The UCB1 controller was used in these experiments. Similar manipulation experiment is shown in Fig. 1d.

**Movie S3** A video showing the particle sorting experiments. Similar experiments were done to obtain the data in the confusion matrix in Fig. 3c.

**Movie S4** Single particle manipulation with bubbles. This video demonstrates the ability of our method to perform a manipulation task even in the presence of air bubbles in the chamber. The controller adapts to the disturbances from the bubbles and successfully guide the particle through the planned path. The experiment shown in the movie was done using the ε-greedy controller."

**Supplementary Note 1: Pseudocode for the multiarmed bandit control algorithms**

In the following, $N = 100$ is the number of discrete frequencies used for the manipulation. In our experiments, the hyper parameters below were chosen as $\epsilon = 0.1$, $\gamma = 0.999$ and $c = 0.001$ pixels (each pixel corresponding to 6.9 μm). These hyper parameters were chosen based on initial exploration as they resulted in successful manipulation. The algorithms are given here in recursive form.

**The ε-greedy control algorithm with decaying memory**

- Parameters: $\varepsilon \in (0,1]$ and $\gamma \in (0,1]$
- Initialization: $w_i(0) = \mu_i(0) = 0$ for $i = 1 \ldots N$
- For each control step $t = 1 \ldots$

1. If any of the frequencies has not been played (i.e. $w_i(t-1) = 0$)
   - $\pi(t) = i$

   Else with the probability $\varepsilon$:

   - $\pi(t) =$ randomly from the range $1 \ldots N$

   Else:

   - $\pi(t) = \arg\max_{i} \mu_i(t-1)$
2. Set $d_k(t)$ as the distance of particle $k$ from its target point (detected using machine vision)
3. Play frequency number $\pi(t)$
4. Set $\tilde{d}_k(t)$ as the distance of particle $k$ from its target point (detected using machine vision)
5. Calculate reward: $r(t) = \sum_k \tilde{d}_k(t) - d_k(t)$
6. Decay weights: $w_i(t) = \begin{cases} \gamma w_i(t-1) + 1 & \text{if } i = \pi(t) \\ \gamma w_i(t-1) & \text{otherwise} \end{cases}$
7. Update mean: $\mu_{\pi(t)}(t) = \mu_{\pi(t)}(t-1) + (r(t) - \mu_{\pi(t)}(t-1))/w_{\pi(t)}(t)$

**The UCB1 control algorithm with decaying memory**

- Parameters: $c$ and $\gamma \in (0,1]$
- Initialization: $w_i(0) = \mu_i(0) = 0$ for $i = 1 \ldots N$
- For each control step $t = 1 \ldots$

1. If any of the frequencies has not been played (i.e. $w_i(t-1) = 0$)
   - $\pi(t) = i$

   Else:
   - $\pi(t) = \arg\max_{\mathrm{i}} \left( \mu_i(t-1) + c\sqrt{\frac{2\log\left(1+\sum_j w_j(t-1)\right)}{w_i(t-1)}} \right)$
2. Set $d_k(t)$ as the distance of particle $k$ from its target point (detected using machine vision, in pixels)
3. Play frequency number $\pi(t)$
4. Set $\tilde{d}_k(t)$ as the distance of particle $k$ from its target point (detected using machine vision, in pixels)
5. Calculate reward: $r(t) = \sum_k \tilde{d}_k(t) - d_k(t)$
6. Decay weights: $w_i(t) = \begin{cases} \gamma w_i(t-1) + 1 & \text{if } i = \pi(t) \\ \gamma w_i(t-1) & \text{otherwise} \end{cases}$
7. Update mean: $\mu_{\pi(t)}(t) = \mu_{\pi(t)}(t-1) + \frac{r(t) - \mu_{\pi(t)}(t-1)}{w_{\pi(t)}(t)}$
